# The prevalence of trichomoniasis and associated factors among women treated at a university hospital in southern Brazil

**DOI:** 10.1371/journal.pone.0173604

**Published:** 2017-03-27

**Authors:** Fabiane Aguiar dos Anjos Gatti, Etienne Ceolan, Fernando Salles Rodrigues Greco, Paula Costa Santos, Gabriel Baracy Klafke, Gisele Rodrigues de Oliveira, Andrea Von Groll, Ana Maria Barral de Martinez, Carla Vitola Gonçalves, Carlos James Scaini

**Affiliations:** 1 Laboratory of Parasitology, Medical School, Federal University of Rio Grande, Rio Grande do Sul, Brazil; 2 Laboratory of Molecular Biology, Medical School, Federal University of Rio Grande, Rio Grande do Sul, Brazil; 3 Obstetrics and Gynaecology Centre, University Hospital, Federal University of Rio Grande, Rio Grande do Sul, Brazil; Massachusetts General Hospital, UNITED STATES

## Abstract

**Background:**

Trichomoniasis is the most prevalent non-viral sexually transmitted disease (STD) in the world; however, it remains a neglected parasitic disease. This study aimed to determine the prevalence of trichomoniasis and its associated epidemiological factors among women treated at a hospital in southern Brazil.

**Methodology/Principal findings:**

A cross-sectional study was performed to determine the prevalence of this infection in women treated at Hospital Universitário (HU) in Rio Grande, Rio Grande do Sul, Brazil, between January 2012 and January 2015. This study consisted a self-administered questionnaire regarding demographic, clinical, and behavioural data and a molecular diagnosis with polymerase chain reaction (PCR) using the TVK3/7 primer set, which was confirmed with sequence analysis. Of the 345 women surveyed, the overall prevalence of *Trichomonas vaginalis* (*T*. *vaginalis*) was 4.1% (14/345). The prevalence rates were 5.9% among pregnant women, 8.5% among HIV-positive women, and 10.1% among HIV-positive pregnant women. The rates for groups with other significant demographic and clinical features were as follows: 6.6% among women with white skin, 12.3% among women with an income below the minimum monthly wage, 7.4% among women with a vaginal pH greater than or equal to 4.6, and 7.9% among women with a comorbid STD. The multivariate analysis confirmed that pregnant women who were HIV-positive (p = 0.001) and had low incomes (p = 0.026) were the most likely to have this infection.

**Conclusions:**

A multivariate analysis confirmed that HIV-positive pregnant women with low incomes were the participants most likely to have trichomoniasis. These results are important because this Brazilian region presents a high prevalence of HIV-1 subtype C, which is associated with greater transmissibility. Additionally, low family income reveals a socioeconomic fragility that might favour the transmission of this STD.

## Introduction

Trichomoniasis is the most prevalent non-viral sexually transmitted disease (STD) in the world, with an annual incidence of 276.4 million cases. However, this STD has been neglected by researchers [1]. Incidence rates can vary according to several factors, such as older age, sexual activity, number of sexual partners, co-infection with other STDs, phase of the menstrual cycle, methods of diagnosis, and socioeconomic status [2,3].

Studies in Latin America show that the prevalence of trichomoniasis is approximately 3.9%, which is higher than the prevalence of Neisseria gonorrhoeae (1.2%) and syphilis (1.1%) [4]. Among pregnant women in Brazil, studies have recorded a prevalence of 7.7% [5]. Few studies have evaluated the incidence of trichomoniasis in men [6].

Low sensitivity has been associated with the laboratory techniques commonly used for diagnosis and epidemiological surveys, such as the direct microscopic examination of the vaginal fluid or *Trichomonas vaginalis* (*T*. *vaginalis*) cultures [7]. Therefore, studies using molecular techniques (e.g., polymerase chain reaction; PCR) to detect the disease and the availability of these techniques can enhance the diagnosis of this parasitosis [8]. Studies performed throughout Brazil show different prevalence rates for trichomoniasis (3.7% to 30%) in different female populations (e.g., prisoners, sex workers, women living in poor communities, women living in rural communities, women treated at gynaecological outpatient clinics, and pregnant women) [9–14]. However, *T*. *vaginalis* cultures, direct microscopic examinations of the vaginal fluid, cytopathology, or combinations of these methods were used for diagnosis in these studies. The current study aimed to determine the prevalence of trichomoniasis and its associated epidemiological factors among HIV-positive and HIV-negative women treated at a university hospital in southern Brazil using PCR.

## Methods

The Dr Miguel Riet Corrêa Jr. Hospital, located in the urban area of the city of Rio Grande, Rio Grande do Sul, Brazil, is a reference centre for health care in the city and for HIV treatment in the region. The hospital reports an annual average of 6.469 hospitalizations, 380.437 consultations, 6.057 surgical procedures, and 488.918 laboratory investigations. Its academic department includes an average of 60 doctors and 50 nurses each year. It also participates in postgraduate and scientific research.

### Type of study and sample calculation

A cross-sectional study was conducted between January 2012 and January 2015 to analyse the prevalence of *T*. *vaginalis* among women treated at the Gynaecology and Obstetrics Service of the Dr Miguel Riet Corrêa Júnior University Hospital (HU) of the Federal University of Rio Grande (FURG). The Health Research Ethics Committee (CEPAS) of FURG approved this study (Opinion 46/2012).

The estimated sample size was calculated based on the population of 90.000 women (aged 10 years or older) from Rio Grande, RS; the expected prevalence of 15% (4–27%) [11,12,14];^,^ a margin of error of 11%; and an attrition rate of 10%. The minimum necessary sample was estimated as 335 women.

### Study participants

A total of 345 women aged 14 years and older agreed to participate in the study. These women were invited to participate during their visit to the Gynaecology and Obstetrics Service of the HU-FURG. An informed consent document was signed by women over 18 years of age or by the individuals legally responsible for girls under 18 years of age. Participation consisted of answering a structured self-administered questionnaire (with closed-ended questions) and authorising a laboratory diagnosis of trichomoniasis and a medical records search.

### Epidemiological questionnaire

The participants answered a self-administered questionnaire that addressed demographic data (age, skin colour, marital status, years of schooling, socioeconomic level, number of births, and number of children), clinical data (symptoms and diagnoses of other STDs), and behavioural factors (age at onset of sexual activity, number of total partners, number of partners [married or dating] over the past 6 months, and use of contraceptives) (S1 File).

### Collection of cervical fluid samples

During the clinical examination conducted by the study team’s gynaecologist, a cervical fluid sample was collected with a VAGISPEC® brush and placed in a tube containing 1 mL extraction buffer (EB; 10 mM Tris-HCl pH 8.5, 1 mM EDTA). The samples were then stored at -70°C in the Laboratory of Molecular Biology of the Interdisciplinary Area of Biomedical Sciences (AICB), Medical School (FAMED), FURG.

### Molecular diagnosis

DNA was extracted from the cervical samples using the Pure Link^®^ Genomic DNA kit (Invitrogen^®^) following the protocol for animal cells. PCR was performed using the TVK3/7 primer set (5’AT TGT CGA ACA TTG GTC TTA CCC TC-3’/5`-TCT GTC CCG TCT TCA AGT ATG C-3`), which amplifies a 300-bp fragment, according to the protocol of Kengne [15] as modified by Crucitti et al. [16]. The reaction conditions consisted of 35 cycles of denaturation at 90°C for 1 minute, annealing at 60°C, and extension at 70°C for 2 min. The amplicon was visualised on 1.5% agarose gel using blue-green loading dye (LGC Biotecnologia^®^) under ultraviolet light.

A 5-μl volume containing 100 fg of *T*. *vaginalis* DNA extracted from cultural ATCC 30236 strain was used in every PCR assay as a positive control. All steps of PCR mix preparation were performed in a laminar flow hood with aseptic techniques. To monitor crossover contaminations, sterile water was included in the DNA extraction procedure and was used as a negative control in the PCR assay. Reaction mixtures without DNA were run in the first PCRs to detect contamination. The DNA extracted from a PCR-negative sample was used as a negative control.

The molecular diagnosis was confirmed by sequencing the PCR-positive samples at ACTGene Molecular Analysis, Ltd. (Centro de Biotecnologia, UFRGS, Porto Alegre, RS, Brazil) using the AB 3500 Genetic Analyser automated sequencer equipped with 50-cm capillaries and POP7 polymer (Applied Biosystems). The sequences were analysed using Bio Edit software and aligned in BLAST to obtain sequence homology with *T*. *vaginalis*.

### Additional laboratory data

The results of the cytopathology test, viral load, and CD4+ T cells counts in HIV-1-positive patients were obtained from their medical records at HU-FURG.

### Statistical analyses

Crude analyses, including the calculation of the prevalence ratio, confidence intervals, and p-value, were performed using SPSS. For each variable, a significant difference of p < 0.05 was adopted, and Fisher’s exact test or the χ^2^-test was used. The sociodemographic, gynaecological, and laboratory data were analysed.

A multivariate analysis was performed after the crude analysis to avoid confounding factors. The Poisson regression method was used to adjust the analysis. Only variables with p < 0.20 or RP > 1.5 were retained in the hierarchical model and compared with more distantly associated levels.

The sociodemographic variables were included on the first level of the hierarchical model, whereas the gynaecological and laboratory variables were included on the second level. All statistical analyses were performed using SPSS.

The limitations of the study were the time between the collection and processing of the sample and the questionnaire responses based on patient recall.

## Results

The TVK3/7 primer set used in the PCR revealed that 4.1% (14/345) of the women treated at HU-FURG were infected with *T*. *vaginalis*. As Table 1 shows, pregnancy and HIV infection increased the prevalence of *T*. *vaginalis* in the sample. In addition, pregnant women who tested positive for HIV (10.1%) were more likely to have trichomoniasis than those who tested negative (3.2%; p = 0.032).

**Table 1:** Bivariate analysis of *T*. *vaginalis* diagnosis in women via polymerase chain reaction (PCR) using the TVK3/7 primer set, considering the variables pregnancy and HIV infection. Gynaecology and Obstetrics Service of the University Hospital of Rio Grande, Rio Grande do Sul, Brazil, January 2012 to January 2015 (n = 345).

	Samples		Positive		*p-value*
Variable	N	(%)	N	(%)	
**Pregnant (HIV-/HIV+)**					0.032*
No	141	(40.9)	2	(1.4)	
Yes	204	(59.1)	12	(5.9)	
**HIV-positive**(Pregnant/notpregnant)					0.009*
No	239	(69.3)	5	(2.1)	
Yes	106	(30.7)	9	(8.5)	
**Type of visit**					0.014*
Prenatal/HIV -	125	(36.3)	4	(3.2)	
Prenatal/HIV+	79	(22.9)	8	(10.1)	
Gynaecological/HIV -	114	(33.0)	1	(0.9)	
Gynaecological/HIV+	27	(7.8)	1	(3.7)	

*Fisher's exact test

The sample had the following composition: 51% over the age of 26, 59.1% pregnant women, 30.7% HIV positive, 63.47% with an income above the minimum wage. No study participant was under the age of 18 years.

Regarding the sociodemographic data, trichomoniasis was more prevalent among women with white skin (6.6%) than among non-white women (2.4%; p = 0.061) and among those with a family income lower than the minimum monthly wage (12.3%) than among those with a higher minimum monthly wage (1.4%; p = 0.000; Table 2).

**Table 2:** Bivariate analysis of the sociodemographic data of women treated at the University Hospital of Rio Grande, Rio Grande do Sul, Brazil. Trichomoniasis was diagnosed using polymerase chain reaction (PCR) with the TVK3/7 primer set. Period between January 2012 and January 2015 (n = 345).

	Samples		Positive		*p-value*
Variable	N	(%)	N	(%)	
**Age**					0.919**
≤ 25 years	164	(48.7)	7	(4.3)	
≥ 26 years	173	(51.3)	7	(4.0)	
**Skin colour**					**0.061**
Non-white	122	(37.1)	5	(2.4)	
White	207	(62.9)	8	(6.6)	
**Education level**					0.363*
≤ 8 years	159	(49.5)	6	(3.8)	
≥ 9 years	162	(50.5)	4	(2.5)	
**Income**					0.000*
< 1 minimum wage	73	(25.0)	9	(12.3)	
≥ 1 minimum wage	219	(75.0)	3	(1.4)	

*Fisher's exact test

**Pearson’s chi-square test

This study found that behaviours and clinical obstetric history were not associated with the diagnosis of trichomoniasis (Table 3).

**Table 3:** Bivariate analysis of the data associated with the behaviour and clinical history of women treated at the University Hospital of Rio Grande, Rio Grande do Sul, Brazil. Trichomoniasis was diagnosed using Polymerase Chain Reaction (PCR) with the TVK3/7 primer set. Period between January 2012 and January 2015 (n = 345).

	Samples		Positive		*p-value*
Variable					
	N	(%)	N	(%)	
**Age at first intercourse**					0.747**
≤ 15 years	181	(52.9)	8	(4.4)	
≥ 16 years	161	(47.1)	6	(3.7)	
**Partner**					0.885**
No	114	(33.9)	5	(4.4)	
Yes	222	(66.1)	9	(4.1)	
**Number of partners in the past 6 months**					0.498*
≤ One	305	(88.4)	12	(3.9)	
≥ Two	40	(11.6)	2	(5.0)	
**Number of pregnancies**					0.227*
≤ One	158	(48.2)	4	(2.5)	
≥ Two or more	170	(51.8)	8	(4.7)	
**Number of births**					0.302*
Nulliparous	118	(36.5)	3	(2.5)	
≥ 1	205	(63.5)	9	(4.4)	
**Contraceptive use**					0.479*
Hormonal	177	(54.8)	6	(3.4)	
None or non-hormonal	146	(45.2)	6	(4.1)	
**Condom use**					0.410**
Yes	155	(46.7)	7	(4.5)	
No	177	(53.3)	5	(2.8)	
**Smoking**					0.217**
No	219	(65.4)	7	(3.2)	
Yes	116	(34.6)	7	(6.0)	

*Fisher's exact test

**Pearson’s chi-square test

A higher prevalence of trichomoniasis was observed among 7.4% of the women with elevated vaginal pH (≥ 4.6) compared with 1.4% of those with lower pH (p = 0.013). In addition, women who reported having other STDs had a higher prevalence of this disease (7.9%) compared with women who were not infected with other STDs (1.4%; p = 0.004; Table 4).

**Table 4:** Bivariate analysis of the laboratory data, clinical signs, and diagnosis of other STDs in women treated at the University Hospital of Rio Grande, Rio Grande do Sul, Brazil. Trichomoniasis was diagnosed using polymerase chain reaction (PCR) with the TVK3/7 primer set. Period between January 2012 and January 2015 (n = 345).

	Samples		Positive		*p-value*
Variable					
	N	(%)	N	(%)	
**Another STD**					0.004*
Yes	114	(34.4)	9	7.9	
No	217	(65.6)	3	1.4	
**Vaginal discharge**					0.131**
Yes	122	(38.0)	8	6.6	
No	199	(62.0)	6	3.0	
**Combined pH**					0.013*
≤ 4.5	148	(55.0)	2	1.4	
≥ 4.6	121	(45.0)	9	7.4	
**Whiff test**					0.504*
Positive	37	(14.8)	2	5.4	
Negative	213	(85.0)	9	4.2	
**Cytopathology**					0.944**
Normal	132	(38.3)	5	3.8	
Inflammatory lesions	109	(31.6)	5	4.6	

*Fisher's exact test

**Pearson’s chi-square test

The multivariate analysis confirmed that among the women in the sample, pregnant women who were HIV positive (RP = 58.05, CI = 5.80–580.62, p = 0.001) and had low incomes (RP = 7.79 CI = 1.27–47.85 p = 0.026) were the most likely to have this infection (Table 5).

**Table 5:** Multivariate analysis of the diagnosis of trichomoniasis in women treated at the University Hospital of Rio Grande, Rio Grande do Sul, Brazil. Trichomoniasis was diagnosed using polymerase chain reaction (PCR) with the TVK3/7 primer set. Period between January 2012 and January 2015 (n = 345).

Variable	N (prevalence %)	95% CIs	*p-value*
**Type of visit**^a^			
Gynaecological/HIV-	1 (0.9)	1.0	
Prenatal/HIV-	4 (3.2)	1.69 (0.25–11.37)	0.586
Gynaecological HIV+	1 (3.7)	4.23 (0.34–51.41)	0.257
Prenatal/HIV+	8 (10.1)	58.05 (5.80–580.62)	0.001*
**Pregnant** ^a^	2 (1.4)12 (5.9)		
No			
Yes			
**HIV positive** ^a^	5 (2.1)	1.0	0.850
No	9 (8.5)	1.12 (0.32–3.98)	
Yes			
**Skin colour** ^b^	5 (2.4)		
White	8 (6.6)	1.0	0.196
Non-white	3 (1.4)	2.30 (0.65–8.13)	
**Monthly income** ^b^	9 (12.3)		0.7660.026*
< 1 minimum wage	2 (1.4)	1.0	
≥ 1 minimum wage	9 (7.4)	1.26 (0.27–5.84)	
		1.07.79 (1.27-	0.466
**Vaginal pH** ^c^		47.85)	
≤ 4.5		1.0	
≥ 4.6		1.76 (0.38–8.17)	

Level of variable in the multivariate analysis

^a^ first

^b^ second

^c^ third

*Fisher's exact test

## Discussion

The *T*. *vaginalis* infection rate of 4.1% in the cervical samples of women treated at a university hospital in southern Brazil matches the rates (3–6%) reported for women treated at gynaecology clinics using molecular diagnostic techniques [17,18].

The higher prevalence of trichomoniasis observed in women who tested positive for HIV compared with those who tested negative was expected. Approximately 6.2% of all HIV-1 infections among women from the USA could be the result of *T*. *vaginalis* infection [19]. However, in Africa, the prevalence of HIV in women infected with *T*. *vaginalis* was 35.8% [20]. Furthermore, previous studies have reported that trichomoniasis is associated with a 1.5- to 3-fold increased risk of HIV infection [21,22] *T*. *vaginalis* infection causes an inflammatory response by recruiting CD4+ T cells and macrophages to the vaginal and cervical mucosa [23]. The punctate mucosal haemorrhages caused by *T*. *vaginalis* may compromise the mechanical barrier of the vaginal flora, thereby favouring HIV-1 infection [24].

The highest prevalence of trichomoniasis was found among pregnant women who tested positive for HIV; these variables were significant in both the bivariate and multivariate analyses. This finding confirms the importance of the co-occurrence of pregnancy and HIV infection. This finding is important because a higher prevalence of HIV-1 subtype C has been reported in the geographic area of the current study, and the clinical course of these infected patients is slow (asymptomatic), thereby favouring the transmission of HIV [25,26]. However, more pregnant women than non-pregnant women were infected with *T*. *vaginalis*, and this difference was significant only in the bivariate analysis. This finding reinforces the importance of the combination of pregnancy and HIV infection as a risk factor for trichomoniasis.

The prevalence of *T*. *vaginalis* infection in pregnant women was similar to that observed in studies conducted in the Brazilian states of Rio de Janeiro (3.7%), Amazonas (5.6%), and Ceará (6.2%). These rates were calculated based on direct examinations of fresh preparations of vaginal fluid [14,27,28]. The rate was also similar to that observed among pregnant women (7.7%) aged 15 to 24 years who were treated at public maternity hospitals across several Brazilian regions [5]. This high prevalence of *T*. *vaginalis* infection among pregnant women compared with non-pregnant women might be explained by the interruption of contraceptive use, an increased frequency of sexual intercourse, hormonal changes, or the increased vaginal pH that occurs during pregnancy [29,30].

The prevalence of *T*. *vaginalis* infection among non-pregnant women (1.4%) was lower than the rates observed in other studies of non-pregnant females aged 12 to 52 years conducted in Ceará (4.1%) [31]; São Paulo, SP (3.2%) [32]; and Minas Gerais (2.6%) [33]. In contrast, the prevalence of *T*. *vaginalis* infection in the current study was similar to that observed in a study conducted in Bahia (1%) [34].

Regarding the sociodemographic data, trichomoniasis was more prevalent among women with family incomes below the monthly minimum wage. These data indicate a socioeconomic fragility that represents a risk factor for trichomoniasis [3]. This variable was also significant in the multivariate analysis.

Women with white skin colour showed a higher prevalence of trichomoniasis than those with a non-white skin colour. This finding does not match those of other studies conducted in Brazil and the USA showing that black people are more likely to have this infection. These studies were performed at a variety of research sites (schools, primary health care clinics, prenatal clinics, and STD clinics) [35–39].

The other sociodemographic data (age and years of education) and risk behaviours (age at first intercourse, having a sexual partner, number of sexual partners, use of hormonal contraception, and condom use) analysed in the present study were not associated with the diagnosis of trichomoniasis.

A vaginal pH level ≥ 4.6 has been identified as a risk factor for trichomoniasis. Brotman et al. [40] revealed that 90% of women with trichomoniasis have a pH level > 4.5, which is a criterion for diagnosing bacterial vaginosis [41], *T*. *vaginalis* infection or both. Doderlein's bacillus is one of the vaginal flora components responsible for the conversion of glycogen to lactic acid, which maintains a vaginal acidity of approximately 3.8 to 4.2 and prevents the development of pathogenic microorganisms. It is known that changes in pH are responsible for bacterial vaginosis (BV); however, an alkaline pH above 4.5 is associated with the presence of Trichomonas [42,43].

Another important aspect is the increase in vaginal pH (approximately 5.5 to 5.8) observed during pregnancy. This increase facilitates protozoan colonisation and therefore explains the higher prevalence of infections observed in pregnant women compared with non-pregnant women. However, other signs or laboratory data associated with trichomoniasis (e.g., vaginal discharge and cytopathologic changes) were not identified in the current study.

In conclusion, pregnant women who are HIV-positive and have a low income were the sample subgroup most likely to have trichomoniasis. These results are important because this Brazilian region is associated with a high prevalence of HIV-1 subtype C, which is associated with greater transmissibility. In addition, low family income represents socioeconomic fragility, which might favour the transmission of this STD.

## Supporting information

S1 File Questionnaire.pdf. (PDF)
