# Supplementary material for: The prevalence of trichomoniasis and associated factors among women treated at a university hospital in southern Brazil
Source: PLoS One. 2017 Mar 27;12(3):e0173604. doi: 10.1371/journal.pone.0173604 (PMC5367685; doi:10.1371/journal.pone.0173604)
Supplement: S1 File — (PDF) [file pone.0173604.s001.pdf]

| The prevalence of trichomoniasis and associated factors among women treated at a University hospital in southern Brazil<br><br>UNIVERSIDADE FEDERAL DO RIO GRANDE, RS<br><br>Patient Category<br><br>(1) Pregnant (2) Pregnant /HIV+ (3) gynecological (4) gynecological /HIV+ | Questionnaire Number<br>______<br>catp __ |
|---|---|

**"QUESTIONS OF THIS QUESTIONNAIRE IS CONFIDENTIAL, THERE IS NOT THE POSSIBILITY OF YOU BE IDENTIFIED. THEREFORE WE COUNT ON YOUR SINCERE".**

| "LET'S TALK ABOUT YOUR PERSONAL DATA" | DO NOT WRITE HERE |
|---|---|
| **1.** What is the color of your skin : (1) White (2) Brown and dark (3) Black | cor __ |
| **2.** How old are you? __ __ years *(complete)* | idad __ __ |
| **3.** You are: (1) Single (2) Married or have companion (3) widow (4) Separated | estaciv __ |
| **4.** Until what grade have you studied? ___ ª of ___ º degree (*already completed)* | serim __<br>graum __ |
| **5.** Do you smoke ? (1) **Yes.** How many cigarettes a day? _______ (2) No (3) Stop smoking | fum __<br>qfum __ |
| **6.** How much Money did the working people from your house earned last month?<br>Person 1: R$ __ __ __ __, __ __<br>Person 2: R$ __ __ __ __, __ __<br>Person 3: R$ __ __ __ __, __ __ | rend1__ __ __ __<br>rend2 __ __ __ __<br>rend3 __ __ __ __ |
| **"NOW LET´S TALK ABOUT SEX, PREGNANCY AND SEXUAL DISEASES"** | |
| **7.** How old where you when you had sex for the first time? __ __ anos | idsex __ __ |
| **8.** With how many people did have sex in your life? ____ | sexv __ __ |
| **9.** With how many people did have sex in the last 6 months? | sexm __ |
| **10**. How many times where you pregnant (births plus abortions)? ___ | gest __ __ |
| **11**. How many birth did you give (parto normal mais cesárea)? ____ | para __ __ |
| **12.** What do you do to avoid getting pregnant? (1) pílula anticoncepcional (2) condom (3) DIU (4) ligadura das trompas (5) Other method. Which? _________________ | faz __<br>qoutro __ |
| **13.** Do you you use condoms during sexual relationships?<br>(1) yes **(2) no.** Which don´t you use? ________________________________________ | usa __<br>porn __ |
| **14.** Have you ever get a sexual tansmissible diseasel?<br>**(1) yes.** Which? ______________________ (2) no (9) I don´t remember | dst __<br>qdst __ |
| **15.** Have ever heard of **Herpes genita**l? (1) yes (2) no (9) I don´t remember | herp __ |
| **16.** Do you know Herpes. How do you get it? ______________________________ | pherp __ |
| **17.** If you know. What does it causes? ______________________________ | cherp __ |
| **18.** Have you ever heard of **Clamídia**? (1) yes (2) no (9) I don´t remeber | clam __ |
| **19.** If you know Clamídia. How do you get it? ______________________________ | pclam __ |
| **20.** If you know Clamídia. What does it causes? ______________________________ | cclam __ |
| **21.** Have ever heard of **Papiloma Vírus Humano (HPV)**?(1) yes(2) no(9) I don´t remeber | hpv __ |
| **22.** If you know HPV. How do you get it? ______________________________ | phpv __ |
| **23.** If you know HPV. What does it causes? ______________________________ | chpv |
| **"NOW LET´S TALK ABOUT CERVICAL CANCER** | |
| **24.** Have ever heard of pre-colon cancer (preventive)?<br>(1) Yes (2) No (9) I don´t remeber | falacp __ |
| **25.** Have you ever did this exam? (1) Yes (2) No (9) I don´t remeber | fezcp __ |
| **26.** Mark what do you believe is relevant to get colon cancer: (multiple choice) believe<br>(1) mother having had cancer (2) sister having had cancer (3) corrimento (4) using DIU<br>(5) Papiloma Vírus Humano (HPV) (6) having had colon wounds (7) Smoking (8) Having many children ( ) Another factor? ______________________ | causa1 __<br>causa2 __<br>causa 3__<br>causa4 __<br>causa 5 __ |

| | |
|---|---|
| **IF YOU ARE NOT PREGNANT, THANKW FOR YOUR PATIENCE.**<br>**IF YOU ARE PREGNANT, PLEASE ANSWER THE FOLLOING QUESTIONS.**<br>**“NOW LET´S TALK ABOUT CORRIMENTO DURING PREGNANCY”** | causa6 __ |
| **35.** Do you have or ever had vaginal corrimento during this pregnancy?<br>(1) Yes, treated (2) Yes, not treated (3) No (9) I don´t remember | gecorr __ |
| **36. IF YES** How many times did youd had corrimento during pregancy? __ __ times<br>(77=durante toda a gravidez; 88=não se aplica; 99=IGN) | gvez __ |
| **37.** Which **color** was these corrimentos?<br>White–yellow: (1) Yes (2) No (9) I don´t remeber<br>Yellow: (1) Yes (2) No (9) I don´t remeber<br>Green: (1) Yes (2) No (9) I don´t remeber<br>Another color: ________________________________________ | gcor1 __<br>gcor2 __<br>gcor3 __<br>gcor4 __ |
| **38.** Did these corrimentos have bad **smell**?<br>(1) Yes, allways (2) Yes, sometimes (3) No (9) I don´t remeber | gcheiro __ |
| **39.** When you were with corrimento, what else did you have?<br>Itching: (1) Yes (2) No (9) I don´t remember<br>Burning during urination: (1) Yes (2) No (9) I don´t remember<br>Pain during sexual relationships: (1) Yes (2) No (9) I don´t remember | gcoc __<br>gard __<br>gdor __ |
| **40.** Have you ever made any treatment for corrimento?<br>**(1) Yes: Which?** (2) No (9) I don´t remember<br>(1) Vaginal cream. Which one and for how long? _____________________<br>(2) Pills. Which and for hou long? _____________________ | gtrat __<br>gcreme __<br>qqcr __<br>gtcr __ __<br>gcomp __<br>gqcomp __<br>gtcomp __ __ |
| **THNK YOY VERY MUCH FOR YOUR PATIENCE!** | |
| **“FOR RESEARCHER USE”**<br>**TEST**<br>**41.** Vaginal pH: ______<br>**42.** Positive Whiff Test: (1) Yes (2) No (9) Not made | ph __ __<br>teste |
| **43.** HIV STATUS(___/____/_____): CD4 ______________<br>Viral Charge ____________ | cd4 _____<br>cv _______ |
| **44. HERPES STATUS**: DNA (PCR): (1) positive (2) negative<br>HERPES tipe: ______ | DNAH __<br>tipo ___ |
| **45. CLAMÍDIA STATUS**: DNA (PCR): (1) positive (2) negative<br>**46.** HPV Genotype: DNA-HPV (PCR): (1) positive (2) negative<br>HPV Genotype: ______<br>HPV Genotype: ______<br>HPV Genotype: ______<br>HPV Genotype: ______ | DNAC __<br>DNAHPV __<br>geno1 ___<br>geno 2___<br>geno 3 ___<br>geno 4 ___ |
| **47.** CITOPATOLOGIC RESULT: (1) Normal (2) Inflammatory ____________<br>(3) low degree injury (HPV and NIC I)<br>(4) high degree injury (NIC II and III and carcinoma “in situ”)<br>(5) Carcinoma invader | cp __ |
